# Impact of Dim Light at Night on Urinary 6-Sulphatoxymelatonin Concentrations and Sleep in Healthy Humans

**DOI:** 10.3390/ijms21207736

**Published:** 2020-10-19

**Authors:** Katarina Stebelova, Jan Roska, Michal Zeman

**Affiliations:** 1 Department of Animal Physiology and Ethology, Faculty of Natural Sciences, Comenius University, Ilkovicova 6, 842 15 Bratislava, Slovak; jan.roska@savba.sk (J.R.); michal.zeman@uniba.sk (M.Z.); 2 Department of Genetics, Cancer Research Institute, Biomedical Research Center, Slovak Academy of Sciences, 845 05 Bratislava, Slovak

**Keywords:** circadian, chronodisruption, light pollution, ALAN, melatonin, 6-sulphatoxymelatonin, aMT6s/creatinine, sleep quality

## Abstract

Artificial light at night can have negative effects on human wellbeing and health. It can disrupt circadian rhythms, interfere with sleep, and participate in the progress of civilisation diseases. The aim of the present study was to explore if dim artificial light during the entire night (ALAN) can affect melatonin production and sleep quality in young volunteers. We performed two experiments in real-life home-based conditions. Young volunteers (*n* = 33) were exposed to four nights of one lux ALAN or two nights of five lux ALAN. Melatonin production, based on 6-sulphatoxymelatonin/creatinine concentrations in urine, and sleep quality, based on actimetry, were evaluated. Exposure to ALAN one lux during the entire night did not suppress aMT6s/creatinine concentrations but did aggravate sleep quality by increasing sleep fragmentation and one-minute immobility. ALAN up to five lux reduced melatonin biosynthesis significantly and interfered with sleep quality, as evidenced by an increased percentage of one-minute immobility and a tendency of increased fragmentation index. Our results show that people are more sensitive to low illuminance during the entire night, as previously expected. ALAN can interfere with melatonin production and sleep quality in young, healthy individuals, and both processes have different sensitivities to light.

## 1. Introduction

Artificial light is an inevitable part of our life, but excessive usage of lighting results in light pollution, which can negatively affect the environment and human health. Artificial light at night (ALAN) can disrupt circadian rhythms of physiological and behavioural processes and participate in the recent burden of civilisation diseases, such as diabetes type 2, obesity, cardiovascular diseases, and cancer [1]. Mechanisms underlying the negative consequences of ALAN on health are still insufficiently understood. Light pollution is an increasing trend [2,3]. Recent measurements have confirmed that 99% of the population in Europe lives in light-polluted areas [4]. Electric street lighting, although much dimmer than indoor lighting, illuminates the outside environment during the night a hundred times brighter than the full moon [5]. In this context, exploring the mechanisms of chronodisruption and establishing threshold values for non-visual effects of light, which desynchronise circadian rhythms and have negative effects on wellbeing and health, is needed.

One mechanism how ALAN can influence different physiological and behavioural rhythms is via suppression of circadian melatonin production, which represents a basis for the light at night hypotheses [6]. Melatonin is an endogenous hormone synthesised in the pineal gland during the night, and its circadian biosynthesis is regulated by the suprachiasmatic nuclei (SCN) of the hypothalamus, which represent the master circadian clocks. They are connected with the pineal gland via the sympathetic part of the autonomic nervous system and control rhythmic melatonin production. Melatonin is synthesized from serotonin following acetylation by arylalkylamine-*N*-acetyltransferase (AA-NAT) and methylation by hydroxyindole-*O*-methyltransferase. Circadian control of melatonin production is exerted through enzyme AA-NAT, which is under circadian control and its activity is directly inhibited by light at night. The synthetized hormone is immediately released and serves as a synchronizing signal for different organs in the body. Since high nocturnal melatonin levels reflect the length of the photoperiod, melatonin can transmit information about environmental conditions to the internal milieu of organisms and can serve as a clock and a calendar ([7,8] for a review). Melatonin receptors are present in different organs, including the brain and SCN ([9,10] for a review). This is important because, in this way, melatonin can feed-back to the central clock and execute its fine-tuning with prevailing environmental conditions.

Melatonin is metabolised very quickly and therefore, its concentrations in biological fluids (plasma, saliva, urine) decline nearly immediately after bright light exposure. Because its concentrations can be easily measured in bodily fluids, melatonin levels are frequently used as a parameter reflecting the effects of light on the circadian rhythms [7,11]. In mammals, including humans, melatonin is metabolised to 6-sulphatoxymelatonin (aMT6s) in the liver and excreted in the urine. Therefore, measurement of aMT6s in the urine represents a useful tool to monitor the stability of the circadian system and acute suppression of melatonin production by light [12,13].

The suppressive effects of ALAN on melatonin biosynthesis have been known for a long time, but the threshold which effectively suppresses melatonin has not been defined [14]. Older studies proved that the human circadian system could be controlled by light, but relatively high intensities were documented. The original chronobiological human studies from the “Andechs bunker” showed that the “normal” 24 h light-dark cycle of 300: 0.1 lux was a weak entraining cue, and social and behavioural rhythmic inputs were more efficient in the entrainment of temperature, cortisol, and melatonin rhythms. Only very bright light (intensity > 2500 lux) was shown to be the strongest entraining agent for human circadian rhythmicity [15]. Later, a well-controlled human study demonstrated that humans could be more sensitive to light than previously expected and light with illuminance less than 100 lux during the early biological night was found to suppress melatonin production and delay its circadian phase [16]. Moreover, a recent study pushed this limit to an even lower range, demonstrating that 2.9 lux can interfere with melatonin production and 6.0 lux can result in 50% melatonin suppression in sensitive individuals [17].

The above-mentioned studies were performed under well-controlled laboratory conditions. However, studies exploring the effects of a low level of ALAN under normal, home-based conditions, are important, but rare. Results from the HEIJO-KYO cohort study in elderly volunteers [18,19] and patients with bipolar diseases [20] demonstrated a strong association between dim ALAN exposure and worsened sleep quality under home-based conditions.

The aim of our study was to determine if low illuminance of ALAN (one and five lux) during the entire night can affect melatonin production evaluated by 6-sulphathoxymelatonin (aMT6s) in the urine of young volunteers and if these conditions can influence sleep quality in home-based conditions.

## 2. Results

### 2.1. ALAN to 1 Lux Exposure

**Figure A1:**
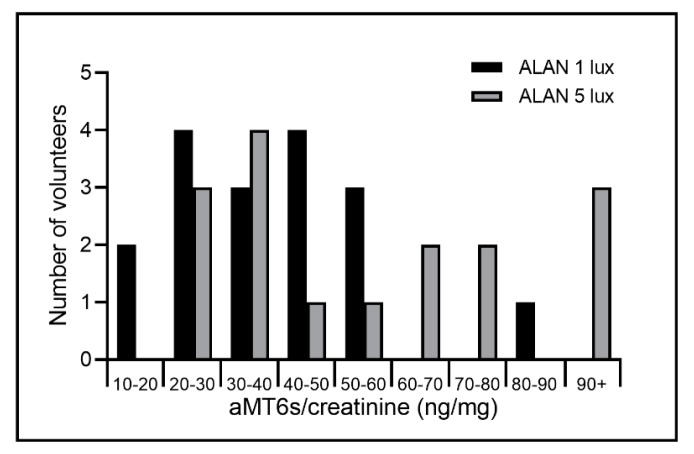
Allocation of volunteers according to the mean amount of aMT6s/creatinine in FMU under control conditions. Black columns are mean control values of volunteers involved in ALAN to 1 lux experiment and grey columns are mean control values of volunteers involved in ALAN to 5 lux experiment.

As expected, the concentration of aMT6s normalised on creatinine in the urine of healthy volunteers was high in the first-morning urine (FMU) and reached basal values in the urine in the middle of the light phase. The concentrations did not differ between the nights before and after ALAN exposure (Table 1). Huge interindividual variability in aMT6s/creatinine concentrations in FMU was recorded among volunteers (Table 1, Appendix A Figure A1).

Sleep quality analysis revealed a higher fragmentation index (*p* < 0.05) and one-minute immobility (*p* < 0.01) during sleep under ALAN 1 lux conditions compared to control nights (paired T-test) (Table 2). The score for one-minute immobility indicates repeated and very short immobility phases during sleep, which results in more interrupted sleep. We found a tendency to more moving minutes (*p* = 0.08) and more immobile phases (*p* = 0.077) during sleep in ALAN 1 lux conditions (Table 2). Thus, all these parameters indicated worsened quality of sleep.

### 2.2. ALAN up to 5 Lux Exposure

**Figure 1:**
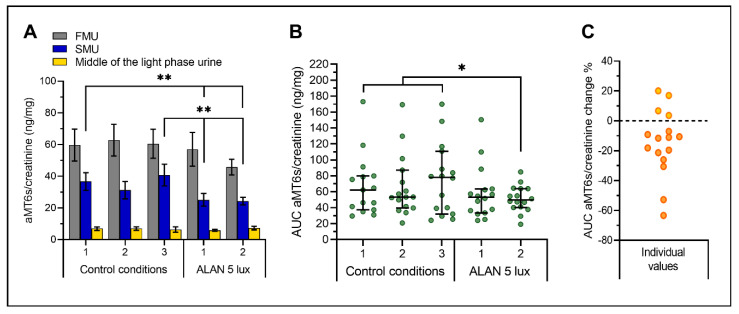
Mean aMT6s/creatinine concentrations (**A**), AUC of aMT6s/creatinine (**B**), and percent of individual aMT6s/creatinine change (**C**) in healthy volunteers (*n* = 16) after three control nights (sleeping in a dark room) and ALAN 5 lux (exposure up to 5 lux during the entire two nights). FMU—first-morning urine, SMU—second-morning urine, urine in the middle of the day collected between 12:00–15:00 hrs, AUC—area under the curve. ** *p* ˂ 0.01, * *p* ˂ 0.05 (ANOVA for repeated measurements with Fisher LSD multiple comparison test). (**A**) The bars represent mean ± SEM, (**B**) Dot plot representing individual AUC values calculated from three urine samples of each individual and median with 95% CI, (**C**) Individual dot plots represent changes in AUC after ALAN exposure, negative values indicating aMT6s/creatinine reduction.

Also, in this trial, aMT6s/creatinine concentrations were high in FMU, decreased in the second-morning urine (SMU), and reached the basal values in the middle of the light phase (Figure 1A). The mean concentrations of aMT6s/creatinine after ALAN 5 lux exposure were not changed significantly in FMU (*p* = 0.214, ANOVA for repeated measures), but were significantly reduced in SMU (*p* = 0.006) after ALAN 5 lux conditions (ANOVA with repeated measures and Fisher LSD post hoc test). Concentrations in the urine collected in the middle of the light phase were low and not affected by ALAN (Figure 1A). The interindividual variability in FMU under control condition is illustrated in Appendix A, Figure A1.

When the area under the curve (AUC) was calculated from three urine samples of each individual on each day of the experiment (Figure 1B), ANOVA with repeated measures revealed significant differences among experimental days (*p* = 0.039). Post hoc analysis specified that on the second day after ALAN 5 lux exposure, concentrations of aMT6s/creatinine were significantly reduced (Figure 1B).

Extensive interindividual variability among volunteers could hide potential individual differences in response to ALAN, and therefore, we calculated the percentage change based on individual AUC of aMT6s/creatinine during control nights. The results demonstrated, that 12 out of 16 subjects (75%) exposed to ALAN 5 lux exhibited aMT6s/creatinine reduction (23.43% ± 5.14% mean ± SEM) (Figure 1C).

Sleep parameters after ALAN 5 lux exposure were changed less than in the ALAN 1 lux trial. We found a significant increase in the percent of one-minute immobility (*p* = 0.037) after the exposure. The fragmentation index showed a tendency to increase (*p* = 0.123) after ALAN 5 lux (Table 3).

## 3. Discussion

In our study, we performed two experiments in real-life home conditions and exposed young volunteers (*n* = 33) to low, environmentally relevant illuminances, either 1 or 5 lux during several nights. We evaluated melatonin production and sleep quality based on urine aMT6s/creatinine levels and actimetry, respectively. Exposure to lower illuminance (one lux) during the entire night did not reduce aMT6s/creatinine concentrations but did worsen sleep quality, evaluated on the basis of the fragmentation index. Higher illuminance (five lux) reduced melatonin biosynthesis and interfered with sleep quality as evidenced by the increased percentage of one-minute immobility and a tendency to the increased fragmentation index. We revealed high inter-individual variability in basal aMT6s/creatinine levels and high variability in their response to ALAN. Our data suggest that in light-sensitive persons, exposure to very low ALAN can interfere with melatonin biosynthesis and sleep quality.

Urine aMT6s/creatinine concentrations correspond to pineal melatonin production and can be used as a marker of entrained circadian system functioning [11]. Melatonin is produced in a circadian pattern with high concentrations during the night time and is involved in the control of circadian rhythms of different physiological and behavioural processes. Among them, the sleep/wake cycle is of key importance because its disruption can deteriorate several other functions [21]. Suppressed melatonin biosynthesis is related to a weakened circadian control of basic physiological processes and may result in several pathologies, such as cancer [22,23], cardiovascular [24] and neurological diseases [20]. Melatonin can mediate its effect via two classes of membrane receptors coupled with G regulatory proteins, which enable its pleiotropic action [25]. Moreover, melatonin was reported to be a direct free radical scavenger and broad-spectrum antioxidant and through this direct mode is able to modulate the redox state and protect against disturbances of different physiological processes [26]. Therefore, the suppression of its night time levels can have negative consequences on health.

In our study, illuminance at a level up to five lux significantly reduced melatonin production, albeit the effect was detected only in the second-morning urine and not in the first one, which is generally used as a marker of the suppressive effects of ALAN on melatonin biosynthesis ([8] for a review). We found the expected continuous decrease of aMT6s during the day, with the highest levels in the first-morning sample, lower in the second-morning sample, and the lowest at the midday urine sample. To the best of our knowledge, this is the first study in which the second urine sample was correlated with the suppressive effects of low ALAN on aMT6s concentrations. Unfortunately, in the experiment with ALAN 1 lux we measured aMT6s only in the first-morning urine. Therefore, we cannot exclude that the melatonin concentrations in the second-morning urine were reduced after exposure to one lux as it was found after five lux. Our data clearly point out that analysis of the first-morning urine might be not sufficient for evaluation of ALAN effects on melatonin production. Concentrations of aMT6s in third urine sample were low, as expected, and they were not related to ALAN exposure.

Our data show that humans are more sensitive to the disrupting effects of dim light on melatonin biosynthesis than previously considered and are in line with recently published findings [17,27]. The results are important because in human experimental studies, exploring the consequences of bright light on melatonin production, an illuminance of approximately 10 lux is frequently used as a safe background light and even stronger lighting was used in older studies [14].

The non-image forming effects of light on the circadian system are mediated by intrinsically photosensitive retinal ganglion cells (ipRGCs). They were found to project to different brain regions [28], including the SCN, the master clock governing circadian melatonin biosynthesis. Among these target structures, the ventrolateral preoptic area (VLPO) of the hypothalamus might be of special importance [29] because it is involved is sleep control [30]. The M1 subtype of ipRGCs integrates rods/cones and melanopsin signals to drive the non-image-forming effects of light. This subtype is very sensitive to dim, scotopic light and can mediate effects of dim light on sleep quality control [31]. Until now, data about the effects of ALAN on VLPO function are lacking, and it is not known if the VLPO sensitivity to ALAN is similar to that of SCN, which is reflected by the suppressed melatonin production. Our results provide indirect evidence that the VLPO could be more sensitive to ALAN than SCN controlled melatonin production, but further extensive research is needed in this field.

Our study showed that very dim light at night could deteriorate sleep quality. An epidemiological study demonstrated a significant association between ALAN exposure (≤5 lux) and sleep disorders in elderly people [18,19] and patients with bipolar disorders [20] in home conditions. Another experimental study reported that exposure to ALAN during sleep at low intensity (up to 10 lux) caused an increase in awakening and shallow sleep in healthy young men [32]. Moreover, a recent correlational study demonstrated an association between the level of outdoor lighting and less favourable sleep patterns and mood and anxiety disorders in US adolescents [33]. A worsening of sleep quality after entire night exposure to one lux in our study pushed the threshold to even lower levels. The absence of melatonin reduction indicates that decreased melatonin is not the causal cause of disturbed sleep quality.

In our study mostly females we included. The intrinsic circadian period is significantly shorter in women than men and circadian rhythms of melatonin and temperature are entrained to an earlier time relative to the nightly sleep/darkness episode in women compared with men [34]. The neurobiological mechanism underlying this sex difference in entrained circadian phase are not known but can have implication for sleep quality and daytime alertness in women. However, experimental studies [17,18,35,36] did not find significant sex differences in response to ALAN.

Our data demonstrated high inter-individual variability of humans towards the disrupting effects of light and are in line with a previous study [17]. In their [17] thoroughly controlled study, 50% suppression of melatonin production was found in the range from six to 350 lux but at the level of 2.9 lux in the most sensitive person. This huge inter-individual variability deserves more research, not only from understanding the potential negative effects of ALAN but also from the point of personalised medicine. The data indicate that evaluation of the negative effects of ALAN at the group level underestimates the real effects because of high variability.

Reasons for the high sensitivity of the circadian system to the disruptive effects of ALAN are not clear, and several possibilities exist. Since our volunteers were recruited by an advertisement, they probably do not represent the general population because their interest in participation might be motivated by their sensitivity to light. Nevertheless, even if they represent a subpopulation of more sensitive people, these data require a consideration. Another reason which can contribute to substantial inter-individual variability in sensitivity to ALAN could be the photoperiodic history of volunteers. Our study was performed under “real-life conditions”, volunteers were mostly students and were asked not to expose themselves to unusual intensity and quality of light before experiments. Exposure to bright light during the previous day can influence the sensitivity of the circadian system to the suppressive effects of light at night [37]. In general, there is a clear change in lifestyle during the last 50 years, and people spend less time in an outside environment exposed to high light intensity. On the contrary, they spend much more time inside during the day exposed to moderate light levels, and during their evening, they are exposed to light from screens of TV, computers, smart-phones, and other devices.

Moreover, in addition to these acute changes, it is necessary to consider the epigenetic consequences of the changed lighting environment. Several animal experiments indicate that exposure of pregnant female mice to unstable light/dark conditions may have long-lasting effects on their progeny [38,39]. In contrast to the short-lasting consequences of acute exposure to ALAN, in utero effects are mediated by epigenetic mechanisms. These may involve hypermethylation of miR17-92 and increased expression of P21 as one of the targets of this miR cluster [38,40]. P21 is recognised as a potent cyclin-dependent kinase inhibitor that facilitates cell-cycle arrest by interacting with different stimuli and acts both as a tumour-suppressor gene and an inhibitor of apoptosis by interacting with various molecules and transition factors [41]. This is one of many other pathways mediating the long-lasting effects of prenatal exposure to ALAN, which should be studied.

Finally, it is necessary to mention that even ALAN is frequently considered as an environmental pollutant [42]. It has different consequences and broader effects in comparison with classical chemical and physical pollutants, which usually affect well defined and narrow targets. This is one of the reasons why the thresholds determining negative effects of ALAN on human health have not been defined yet.

## 4. Materials and Methods

### 4.1. Volunteers

In the study, 39 young volunteers were recruited (31 women, 8 men) and included in two independent experiments. Six volunteers (3 women, 3 men) were excluded because of not keeping the experimental schedule, missing samples, or very low or abnormal aMT6s profile in the urine. The volunteers were University students and employees recruited per personal interview or advertisement on social networks. The average age of the volunteers was 24.0 ± 0.6 year (± SEM), BMI was in a normal range for this age group (22.6 ± 0.7; mean ± SEM). All volunteers were healthy and not taking any medications except oral contraceptive. Shift work or traveling across time zones was an obstacle for study participation. No volunteers reported suffering from any sleep disorders.

Before the study, all volunteers completed a circadian type questionnaire [43]. A score of 65 or more represented a morning chronotype, and a score 45 or less, an evening chronotype. There were seven morning chronotypes, 16 neutral chronotypes, and 10 evening chronotypes among the volunteers in the experimental groups. All the volunteers were asked to maintain a regular sleep/wake schedule at least two weeks before the beginning of their study part.

Both experiments were organised in Bratislava, Slovak Republic (48°08′38″ N; 017°06′35″ E) during the short photoperiod in months January to March before the daylight-saving time change.

### 4.2. Ethical Considerations

All volunteers provided a signed written informed consent prior to the beginning of the study. The study conformed to the standards of the Declaration of Helsinki and international ethical standards. The study protocol was approved by the Ethical Committee at the Faculty of Natural Sciences, Comenius University Bratislava, Slovak Republic (15.11.2019, ECH19003). Volunteers were financially compensated for their participation in the study.

### 4.3. Study Design

Two independent home-based experiments were performed to test the entire night ALAN impact on melatonin and sleep in human. During both parts of the experiment, volunteers kept their standard life regime but were not allowed to nap. The daily regime and sleep during both experiments were monitored by the wrist accelerometer Actiwatch AW4. Moreover, volunteers also kept a sleep diary, and the sleep start and sleep end were set according to their actigraphy records and sleep diary.

Over each part of the study, volunteers were asked to use a blue light screen saver application from 18:00 hrs in the evening. It changed the intensity and colour temperature of the display, depending on the phase of the day, on electronic devices with LED display (cell phones, tablets, monitors).

#### 4.3.1. Experiment 1—ALAN to 1 Lux Exposure

In this experiment, 17 volunteers (15 women, two men) were included. The experimental trial lasted for seven nights. The control portion lasted for three nights, and the ALAN to one lux part for four consecutive nights. During the control conditions, volunteers slept in a completely dark bedroom. Windows were covered with curtains or jalousies to avoid artificial lighting from the outside environment. During light exposure nights, volunteers were exposed to the light of one lux while sleeping.

Each day of the experiment, urine samples were collected. The first urine sample was taken immediately after waking up (the first-morning urine). The urine sample in the middle of the light phase of the day was collected between 12:00–15:00 hrs. The samples were stored at −18 °C until analysis.

#### 4.3.2. Experiment 2—ALAN up to 5 Lux Exposure

**Figure A2:**
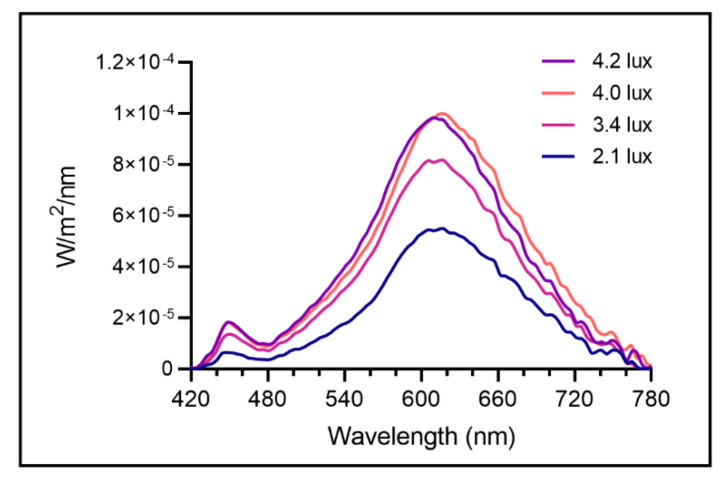
The spectral power distribution across wavelengths of the LED light source used for ALAN 5 lux illumination. Illuminances were measured in different directions on the position of the volunteer’s head during sleeping. Peak wavelength ~610 nm. Measured with spectrophotometer CL-500A (Konica Minolta Sensing Europe BV, Germany).

In the second experiment, 16 volunteers (14 women, two men) were included. The experimental trial lasted for five nights, three control nights and two light contamination nights. During control nights, volunteers slept in a dark bedroom, and during the light contamination nights, the bedrooms were illuminated with ALAN to five lux at the position of the volunteer’s head in bed (Figure A2).

After each experimental night, volunteers provided urine samples. The FMU was taken immediately after waking up, SMU was taken approximately 2 h after waking time. The urine sample in the middle of the light phase of the day was collected between 12:00–15:00 hrs. The samples were stored at −18 °C until analysis.

### 4.4. Lighting Conditions

The illuminance during ALAN to 5 lux was set up and measured at the beginning of ALAN with a spectrophotometer CL 500A (Konica Minolta, Japan). The illuminance was measured at the position of the volunteer’s head in the bed by sleeping in four directions—left, right, up to the ceiling, and towards to the feet (Figure A2).

The illuminance to 1 lux was induced by artificial light from the outside environment by rolling up the jalousies or curtains on the bedroom window. If this was not enough, illuminance through the open door from an adjacent room or corridor was used.

For the illumination to 5 lux, lamps with a commercially available LED light bulb (LED Superstar, Osram) were used. The colour temperature of the used LED was 2700 K, λmax 607 nm (Figure A2). The light source was set up so that at the position of the volunteer’s head while in the bed maximum luminance five lux was measured. During the study, the use of multimedia devices (e.g., cell phones, computers, and televisions) in the evening was not limited or prohibited. The volunteers were not instructed regarding morning light exposure.

### 4.5. Sleep Assessment

During both experiments, the sleep-wake pattern and objective sleep quality were monitored by a wrist accelerometer (Actiwatch AW4, CamNtech Ltd., Cambridgeshire, UK). The accelerometer was placed on the non-dominant hand of the volunteer and worn while sleeping. The epochs of data recording were set to 1 min. This approach was previously used and validated in our study [44].

The sleep quality was evaluated with the Actiwatch sleep analysis program provided by CamNtech Ltd., Cambridgeshire, UK. The used sleep-wake scoring algorithm was validated against polysomnography in adults, and a high correspondence (over 90%) between the two methods was reported [45]. Actimetry is considered a valid and reliable non-invasive method for measuring sleep quality in healthy human studies [46]. The epoch is scored as sleep unless the activity exceeds a certain threshold, which in turn is determined by the selected sensitivity.

The following actigraphy parameters were evaluated in this study: time in bed, assumed sleep, actual sleep time, actual sleep (%), actual wake time, actual wake (%), sleep efficiency, sleep latency, sleep bouts, wake bouts, mean sleep bout time, mean wake bout time, immobile mins, immobile time (%), moving mins, moving time (%), no of immobile phases, mean length immobility, one Min immobility (%), total activity score, mean activity score, mean score in active periods and fragmentation index. An elevated fragmentation index is an indicator of restlessness during the sleep period. The score for one-minute immobility is also an indicator of restless sleep because more very short immobility phases during sleep indicate more interrupted sleeping period.

### 4.6. Melatonin Measurement

The concentrations of aMT6s were determined by radioimmunoassay using a commercially available kit (Stockgrand LTD., Guildford, UK). The assay was performed according to the manufacturer’s instructions using a method adapted for iodinated aMT6s [44,47]. The samples were measured within nine assays. Each sample from one volunteer was analysed within the same assay. The mean intraassay coefficient was 11.6%. The mean interassay coefficient was 22.8%, 16.7% and 14.2% for low, medium, and high-quality control sample, respectively.

The concentrations of aMT6s were normalised against creatinine. The creatinine in human urine was determined by an enzymatic method for quantitative in vitro determination of creatinine in human serum, plasma, and urine (CREA Enz 204, Erba Lachema, Brno, Czech Republic). The samples were treated according to the manufacturer’s instruction, and the absorbance was measured by 492 nm on a spectrophotometer (Apollo LB 913, Berthold Technologies GmbH & Co., Bad Wildbad, Germany).

### 4.7. Statistical Analysis

Results are given as aMT6s/creatinine (ng/mg). Refer to mean ± SEM or AUC. Data were statistically analysed using SigmaPlot 12.5. Data were first assessed for normality with the Shapiro-Wilk normality test. Parametrically distributed data were statistically evaluated according to ANOVA for repeated measurements with the Fisher LSD post hoc test when significant. The non-parametric data were log10 transformed.

The percentual changes of AUC aMT6s/creatinine reduction were calculated for each volunteer individually. The mean aMT6s/creatinine concentration under control conditions was considered as 100%. The percentage change after ALAN 5 lux (mean AUC after ALAN 5 lux) was calculated.

The activity data during the sleep period were automatically analysed with the software Actiwatch Activity & Sleep Analysis 7.

## 5. Conclusions

Our results show that even a low intensity of dim light during the entire night can interfere with sleep quality and melatonin production in young people. The study reveals a need for more intensive research of ALAN on disturbances of physiological processes and health regarding light quality, duration of exposure, and light intensity in relation to the substantial inter-individual differences among people.

## Figures and Tables

**Table 1:** Descriptive statistics of aMT6s/creatinine concentrations in urine of healthy volunteers under control and ALAN 1 lux conditions.

Conditions	Samples	Mean aMT6s ng/mg Creatinine *n* = 17 Each	SEM	Minimum Value ng/mg	Maximum Value ng/mg
Control (3 nights)	First morning urine ^1^	39.75	±2.69	13.30	97.16
	Middle of the light phase urine ^2^	5.69	±0.73	0.98	29.37
ALAN 1 lux (4 nights)	First morning urine ^1^	42.22	±2.50	10.69	107.69
	Middle of the light phase urine ^2^	5.52	±0.72	0.74	43.93

^1^ First morning urine collected immediately after waking up, ^2^ urine collected in the middle of the day (between 12:00–5:00).

**Table 2:** Parameters of sleep quality in young volunteers exposed to ALAN 1 lux during the entire sleeping period, *n* = 17.

	Control Nights Mean ± SEM	ALAN 1 Lux Mean ± SEM	*p* ^1^
Time in bed	7:43 ± 0:14	8:07 ± 0:11	0.080
Assumed sleep	7:23 ± 0:14	7:45 ± 0:10	0.086
Actual sleep time	6:21 ± 0:12	6:39 ± 0:09	0.069
Actual sleep (%)	86.09 ± 1.14	85.92 ± 1.00	0.737
Actual wake time	1:02 ± 0:05	1:06 ± 0:05	0.269
Actual wake (%)	13.91 ± 1.14	14.08 ± 1.00	0.735
Sleep efficiency	82.38 ± 1.12	82.11 ± 1.18	0.752
Sleep latency	0:15 ± 0:02	0:16 ± 0:02	0.655
Sleep bouts	27.25 ± 1.75	28.89 ± 1.42	0.159
Wake bouts	27.27 ± 1.73	28.91 ± 1.41	0.156
Mean sleep bout time	0:15 ± 0:01	0:14 ± 0:00	0.312
Mean wake bout time	0:02 ± 0:00	0:02 ± 0:00	0.822
Immobile mins	375 ± 12	391 ± 10	0.117
Immobile time (%)	84.71 ± 1.30	84.06 ± 1.31	0.129
Moving mins	68.09 ± 6.47	74.45 ± 6.23	0.080
Moving time (%)	15.29 ± 1.30	15.94 ± 1.31	0.129
No of immobile phases	42.26 ± 2.71	45.32 ± 2.48	0.077
Mean length immobility	9.57 ± 0.7	9.29 ± 0.65	0.324
One Minute immobility	7.50 ± 0.91	8.84 ± 1.03	0.003 **
One Min immobility (%)	16.84 ± 1.33	18.71 ± 1.44	0.073
Total activity score	7480 ± 838	8295 ± 950	0.233
Mean activity score	16.77 ± 1.79	17.71 ± 2.00	0.355
Mean score in active periods	112 ± 9	112 ± 9	0.974
Fragmentation index	32.13 ± 2.49	34.66 ± 2.68	0.042 *

^1^ Paired T-test. * *p* < 0.05; ** *p* < 0.01.

**Table 3:** Parameters of sleep quality in young volunteers exposed to ALAN 5 lux during the entire night, *n* = 15.

	Control Conditions Mean ± SEM	ALAN 5 Lux Mean ± SEM	*p* ^1^
Time in bed	7:49 ± 0:09	8:08 ± 0:08	0.083
Assumed sleep	7:42 ± 0:09	8:01 ± 0:08	0.088
Actual sleep time	6:44 ± 0:09	7:00 ± 0:09	0.172
Actual sleep (%)	87.10 ± 1.10	86.83 ± 1.47	0.771
Actual wake time	0:57 ± 0:05	1:02 ± 0:07	0.341
Actual wake (%)	11.94 ± 1.10	12.33 ± 1.45	0.671
Sleep efficiency	85.87 ± 1.16	85.43 ± 1.43	0.654
Sleep latency	0:05 ± 0:01	0:05 ± 0:01	0.496
Sleep bouts	26.84 ± 1.83	28.07 ± 2.60	0.405
Wake bouts	26.87 ± 1.81	27.93 ± 2.66	0.497
Mean sleep bout time	0:16 ± 0:01	0:17 ± 0:02	0.586
Mean wake bout time	0:02 ± 0:00	0:02 ± 0:00	0.525
Immobile mins	410 ± 9	427 ± 10	0.133
Immobile time (%)	88.41 ± 0.83	88.23 ± 1.21	0.812
Moving mins	51.60 ± 3.86	54.40 ± 6.03	0.469
Moving time (%)	10.64 ± 0.85	10.80 ± 1.22	0.836
No of immobile phases	38.03 ± 2.55	39.33 ± 3.56	0.584
Mean length immobility	11.36 ± 0.88	11.93 ± 1.21	0.499
One Minute immobility	4.33 ± 0.63	5.77 ± 1.04	0.074
One Min immobility (%)	10.09 ± 0.99	12.63 ± 1.42	0.037 *
Total activity score	8035 ± 1444	7657 ± 875	0.792
Mean activity score	16.72 ± 3.05	15.33 ± 1.76	0.645
Mean score in active periods	159 ± 32	145 ± 13	0.659
Fragmentation index	21.23 ± 1.77	24.00 ± 2.52	0.123

^1^ Paired T-test. * *p* < 0.05.

## Abbreviations

FMU	First-morning urine
SMU	Second-morning urine
ALAN	Artificial light at night
SCN	Suprachiasmatic nucleus
ipRGCs	Intrinsically photosensitive retinal ganglion cells
VLPO	Ventrolateral preoptic area
M1	Subtype of ipRGC
AUC	Area under the curve
CI	Confidence interval
AA-NAT	Arylalkylamine-*N*-acetyltransferase
